# Investigation of 2-Mercapto-1-Methylimidazole as a New Type of Leveler in Wafer Electroplating Copper

**DOI:** 10.3390/ma18071622

**Published:** 2025-04-02

**Authors:** Tong Tan, Renlong Liu, Lanfeng Guo, Zhaobo He, Xing Fan, Rui Ye, Changyuan Tao

**Affiliations:** 1 School of Chemistry and Chemical Engineering, Chongqing University, Chongqing 400044, China; 202218131179@stu.cqu.edu.cn (T.T.); guolf@sinophorus.com (L.G.); foxcqdx@cqu.edu.cn (X.F.); taocy@cqu.edu.cn (C.T.); 2 Hubei Sinophorus Electronic Materials Co., Ltd., Yichang 443007, China; zhaobo_he@sinophorus.com (Z.H.); pantheon_ye@sinophorus.com (R.Y.)

**Keywords:** chip copper interconnection, wafer plating, leveler

## Abstract

Through-Silicon-Via (TSV) technology is of crucial importance in the process of defect-free copper filling in vias. In this study, the small molecule 2-mercapto-1-methylimidazole (SN2) is proposed as a new leveler. It enables bottom-up super-filling of blind vias without the need for inhibitors. Atomic force microscopy (AFM), X-ray diffraction (XRD), and XPS were employed to characterize the surface morphology, crystal structure, and adsorption properties of copper crystals in these systems. Meanwhile, by means of electrochemical measurements, the inhibitory effect of the leveler SN2 on copper ion deposition and the synergistic effect between SN2 molecules and other additives were investigated. The LSV test indicated that additive SN2 inhibited copper electrodeposition after being added to the plating solution, and this inhibitory effect enhanced with increasing SN2 concentration. In the actual plating wafer test (1 ASD plating for 1 min, 5 ASD plating for 5 min, and 10 ASD plating for 1 h), the best plating effect was achieved at 3 ppm, which verified the conjecture of the galvanostatic measurements. Moreover, XPS and computer simulations revealed that SN2 could be adsorbed onto the copper surfaces. This work will inspire the discovery of new effective levelers in the future.

## 1. Introduction

The advancing electronic information industry and increasing 3D packaging integration demands [1,2] have spurred the use of TSV technology, which enhances performance by enabling vertical conduction between chips, reducing transmission distances [3,4,5]. Electroplated copper is favored for TSV deposition due to its reliability, productivity, and low cost [6,7]. However, uneven current density during plating can cause defects like voids, impacting TSV performance [8,9]. Additives such as Cl⁻, accelerators, inhibitors, and levelers are used in the electroplating solution to enable bottom-up Cu deposition and prevent defects [10,11,12].

Levelers, often nitrogenous heterocyclic compounds or quaternary ammonium salts [13,14], operate under the convection-dependent adsorption (CDA) theory, which posits that they inhibit copper deposition in high-current-density regions through positive charges or specific functional groups, promoting bottom-up copper growth [10,15,16]. According to the convection-dependent adsorption (CDA) mechanism, the low and high rotation speeds of the rotating disk are respectively used to simulate the low-current region at the bottom of the blind hole and the high-current region on the surface of the blind hole. The potential difference (Δη, Δη = η (low rotation speed) − η (high rotation speed)) obtained from tests at two different rotation speeds can be used as an important parameter to evaluate the filling ability of the electroplating solution. If the Δη value of the additive is positive, the electroplating solution may exhibit good filling performance [17,18,19].

Designing an optimal leveler for an effective filling performance is essential. Current research focuses on dye-based levelers like JGB and DB [18,20], which are complex, costly, toxic, and often require inhibitors, highlighting the need for affordable, low-toxicity, stable, and effective levelers in electroplating.

2-Mercapto-1-methylimidazole, an inexpensive, low toxic organic compound with sulfur and nitrogen, shows strong coordination with copper. It was added to the copper plating solution to assess its impact on copper deposition in blind vias. Various techniques, such as cyclic voltammetry, cathodic polarization, galvanostatic measurements, X-ray photoelectron spectroscopy (XPS), quantum chemical calculations, molecular dynamics simulations, atomic force microscopy, and X-ray diffractometry, have been employed to study the interactions between the leveler, other additives, and the copper surface, as well as the effects on the Cu-filled surface topography and structure.

## 2. Experimental

### 2.1. Electrochemical Measurements

**Figure 1:**
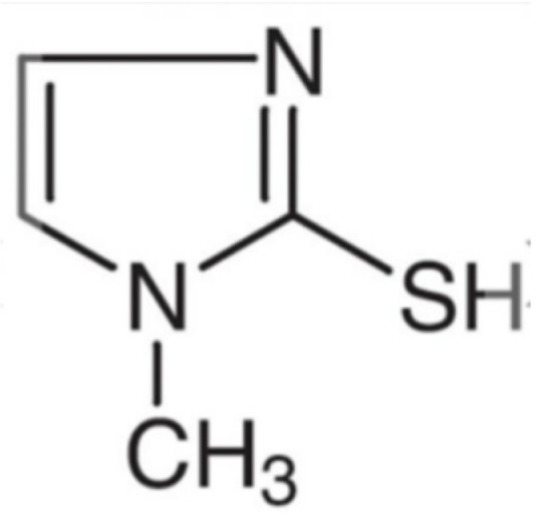
Molecular structure of the leveler: 2-mercapto-1-methylimidazole (SN2).

In this experiment, the CHI760C electrochemical workstation and rotating disk electrode were selected to carry out electrochemical tests. The system was a three-electrode system. The working electrode was a platinum rotating disk electrode, the counter electrode was a phosphor–copper electrode, and the reference electrode was a mercury sulfate electrode. All electrochemical experiments were carried out at room temperature. The voltage scan range of the cyclic voltammetry (CV) experiment was from 0.4 V to −0.8 V, and the scan rate was constantly 50 mV/s. The voltage scan range of the cathodic polarization curve was from 0 V to −1 V, and the scan rate was constantly 10 mV/s. The galvanostatic measurement (GM) was carried out at a current density of 2 A/dm^2^, and various additives were continuously added during the measurement. The Virgin Makeup Solution (VMS) used in the experiment consisted of 220 g/L of CuSO_4_ 5H_2_O (99%, AR) and 50 g/L of H_2_SO_4_ (98%, AR). In addition, the chloride ion (37% HCl, AR) concentration in all experiments was set at 50 ppm. The accelerator was bis(3-sulfopropyl) disulfide (SPS) (99%, AR), and the leveler was 2-mercapto-1-methylimidazole (SN2, 98%, AR, see Figure 1).

### 2.2. Electrodeposition

With the 2 × 2 cm wafer slice being used as the cathode and the phosphorus-containing copper plate being used as the anode, and with the magnetic stirring speed being set at 400 rpm, direct current was applied to the cathode and anode, and 1 ASD was plated for 60 s, followed by 5 ASD for 300 s, and then 10 ASD for 1 h. The plated wafers had micropore depths of 90 μm and diameters of 200 μm. After electroplating, they were polished to the hole cross-section with a grinding and polishing machine and observed under a metallographic microscope.

### 2.3. Characterization

Soaking the brass sheet in ethanol for 5 min to remove the contaminants on it, it was then rinsed with deionized water to ensure the contaminants were completely removed. The copper sheet was used as the electroplating cathode and the phosphorus-containing copper plate as the anode. The sample was plated at a current density of 2 ASD for 10 min to obtain the test piece. The sample was removed with tweezers, its surface rinsed with deionized water, and then dried. This is the way to prepare samples for AFM, SEM, and XRD testing. The sample surface roughness was examined using an Agilent 5500 Atomic Force Microscope (AFM). The surface morphology of the plated samples in different solutions was observed with an S-4800 scanning electron microscope (SEM). A D8 ADVANCE X-ray diffraction spectrometer (Bruker, Ettlingen, Germany) was employed to determine the preferential orientation of the electrodeposited copper layers. XPS was tested using a 1486.60 eV Al Ka source; the test samples were soaked in the bath for 3 h, then rinsed with ultrapure water, dried, and corrected for all XPS results with the C1s (284.8 eV) peak as the reference peak. All experiments were performed at room temperature.

### 2.4. Theoretical Calculation

The optimized molecular structure of 2-mercapto-1-methylimidazole was obtained and quantum chemical calculations were carried out using the DFT theory and the DMol3 module (BLYP model) in Materials Studio. The highest occupied molecular orbital (HOMO), the lowest unoccupied molecular orbital (LUMO), and the energy gap value (∆E = E_LUMO_ − E_HOMO_) of 2-mercapto-1-methylimidazole were obtained. The Forceite module of the Materials Studio 2019 software with the COMPASS force field was used to simulate the molecular motion behavior of 2-mercapto-1-methylimidazole on the Cu (111) crystal plane. The size of the simulation box was 25.6 × 25.6 × 60 nm^3^, which contained one 2-mercapto-1-methylimidazole molecule, one chlorine atom, and 500 water molecules. The simulation time was 500 ps, the simulation step was 1 fs, and the temperature was 298 K. The interaction energy is calculated by the formula E_Adsorption_ = E_Total_ − E_Cu_ − E_Leveler_. In the formula, E_Cu_ is the energy of the copper crystal, E_Leveler_ is the free energy of the 2-mercapto-1-methylimidazole molecule, and E_Adsorption_ is the interaction energy between copper and the leveler molecule [12,21].

## 3. Results and Discussion

### 3.1. Electrochemical Analysis

**Figure 2:**
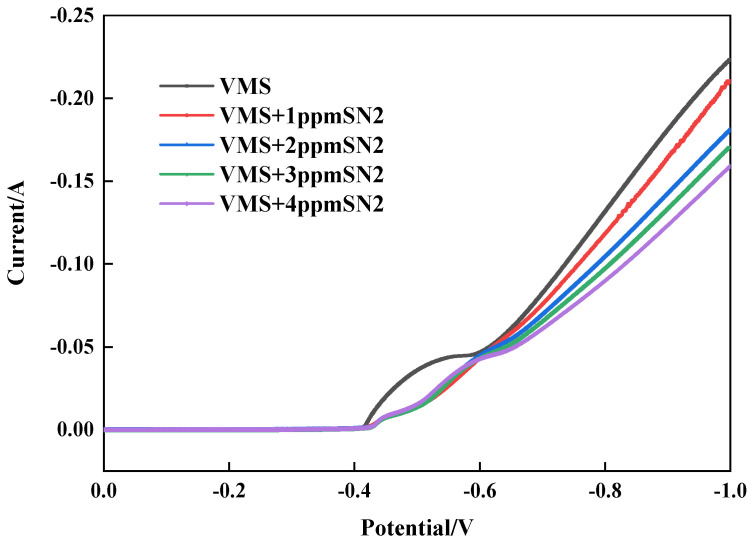
Cathodic curves for different concentrations of SN2.

During the electrodeposition process, copper ions (Cu^2^⁺) obtain electrons from the electrolyte, are reduced to metallic copper, and deposit on the surface of the cathode. This reaction is the core step of copper electroplating, and this process is significantly affected by the additives and their concentrations [8,16]. Through cathode polarization tests with different concentrations of the leveler, its influence on copper deposition was studied (see Figure 2). After adding the leveler SN2, the copper deposition potential shifted in the negative direction. Moreover, the higher the concentration, the more negative the potential became. This indicates that the leveler increases the cathodic polarization by adsorbing on the copper surface, thereby inhibiting copper deposition.

To research the influence of the leveler on the bottom and surface copper deposition behavior during blind hole filling, galvanostatic measurements were conducted at 100 rpm and 1000 rpm to analyze the effect of different concentrations of the leveler on the potential.

**Figure 3:**
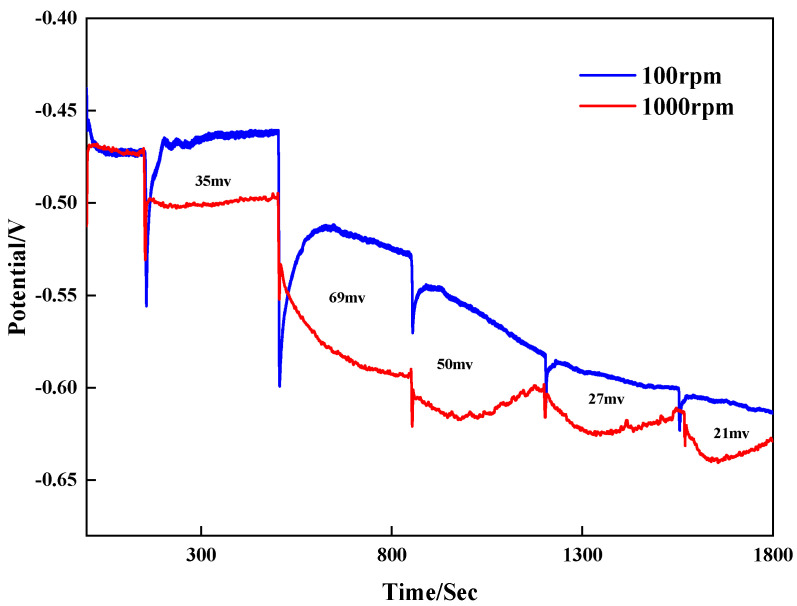
Galvanostatic measurements with different concentrations.

In the plating solution with VMS + 50 ppm Cl⁻ + 1 ppm SPS, 1 ppm SN2 was added at 350 s intervals at a current size of 2 ASD. The corresponding results are shown in Figure 3. With the addition of SN2, the potential gradually shifted negatively, indicating that the leveler enhanced the plating solution’s polarization. The potential difference Δη values of different concentrations of SN2 at high and low rotational speeds are positive, showing an obvious convective adsorption effect. This indicates that strong convection helps the leveler SN2 inhibit copper electrodeposition and implies that its adsorption capacity at the pore opening is stronger than that inside the pores. The potential difference (100–1000 rpm) is larger at 2–3 ppm, so it is expected that the actual plating results are better when the SN2 concentration is 2–3 ppm.

**Figure 4:**
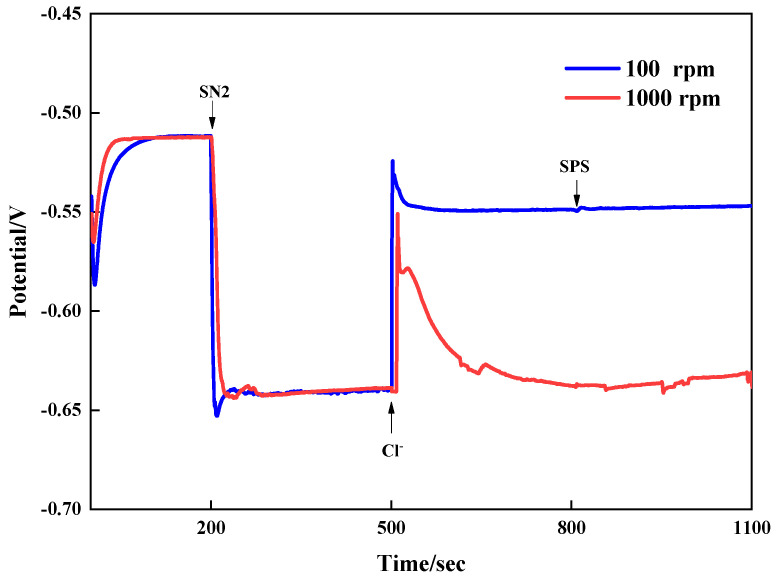
Galvanostatic measurements with additives added at different times.

To analyze the interaction of the leveler SN2 with other additives, galvanostatic measurements were conducted by adding the additives separately at different times. The results are shown in Figure 4. The copper deposition potential was approximately −0.51 V with only the base bath VMS. It shifted sharply negative to −0.643 V when 3 ppm SN2 was added at 200 s. At this time, the high and low rotational speeds had little impact on the system’s potential, indicating that the ability to inhibit copper electrodeposition was not affected by convective strength when only SN2 was present. Interestingly, when Cl- was added to the plating solution, the potential rose to −0.549 V at low speed, with a positive shift of 90 mV. At high speed, adding chloride ions caused the potential to rise and then rapidly fall back to the original potential. At this point, there was a large potential difference between high and low speeds, showing an obvious CDA effect. That is, the inhibitory effect of SN2 on copper was significantly affected by the concentration of chloride ions, being strong at a high chloride ion concentration and weak at a low one.

**Figure 5:**
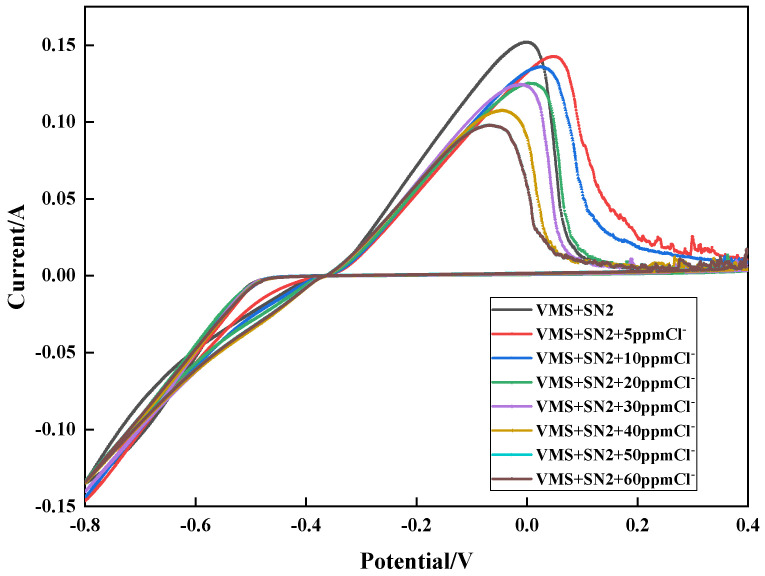
Cyclic voltammetry curves with different chloride concentrations.

To explore the effect of the chloride ion concentration on the inhibition effect of SN2, cyclic voltammetry tests were conducted. The results are shown in Figure 5. When the plating solution contained no chloride ions, the maximum copper stripping peaks were obtained in the test. As the chloride ion concentration increased, the copper stripping peaks became smaller, indicating that the inhibition effect of SN2 on copper electrodeposition is significantly affected by the chloride ion concentration and that the presence of chloride ions enhances its inhibition effect on copper electrodeposition.

### 3.2. Electrodeposition Analysis

**Figure 6:**
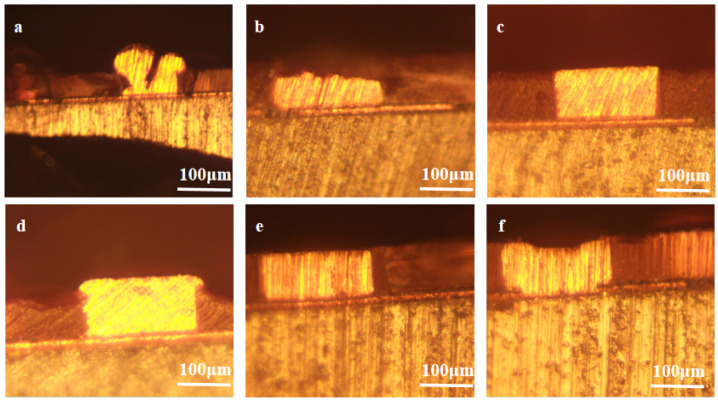
Electroplating effect diagrams of SN2 at different concentrations in the micro-holes of actual wafers. From left to right (**a**–**f**), the SN2 concentration increases gradually from 0 ppm to 5 ppm.

Based on the results of the electrochemical analyses, SN2 is predicted to be an effective leveler for wafer overfilling. Actual filling tests were conducted using electroplating solutions with different concentrations of levelers, and the copper filling results are shown in Figure 6. The plating solution was VMS + 50 ppm Cl^−^ + 1 ppm SPS, and the SN2 concentration increased gradually from 0 ppm to 5 ppm from a to f. When the plating solution contained no leveler, there were obvious defects in the plated wafers and the filling effect was poor. When 1 ppm SN2 was added, there were still defects in the filling of blind vias, perhaps because the concentration of the leveler in bath a was insufficient to effectively inhibit copper deposition on the surface. When the SN2 concentration was 2–3 ppm, the wafer was completely filled with no vias having defects and good flatness. As the SN2 concentration continued to increase, the flatness increased. The area in the middle of the hole was not completely filled and levelled off at a concentration of 5 ppm. This condition is presumed to be triggered by a high levelling agent concentration. Excessive concentrations caused diffusion from the mouth of the SN2 pore to the center region of the pore, thus interfering with the proper functioning of the accelerator.

### 3.3. Characterization Analysis

**Figure 7:**
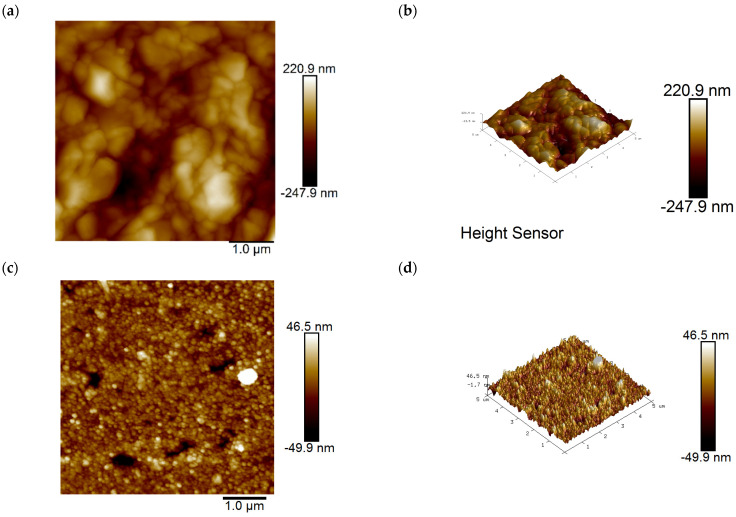
(**a**,**b**) Two-dimensional and 3D height images of the copper surface obtained by electroplating in an SN2-free solution; (**c**,**d**) two-dimensional and three-dimensional height images of the copper surface obtained by electroplating in a solution containing 3 ppm SN2.

Using atomic force microscopy (AFM) from Agilent Technologies Co., Ltd. (Santa Clara, CA, USA), two-dimensional (2D) and three-dimensional (3D) images of the coating surface before and after the addition of SN2 were obtained. It can be clearly and intuitively seen that SN2 has improved the copper surface morphology. In Figure 7a,b, without adding SN2, electroplating results in plated surfaces with grooves and an uneven rough structure, having an average roughness (Ra) of 46 nm. When 3 ppm SN2 was added, the plated layer’s roughness was significantly improved, with the average roughness (Ra) dropping to 8.2 nm, 82.2% lower than before. This shows that SN2 can be effectively adsorbed on copper, changing the surface morphology of the coating.

**Figure 8:**
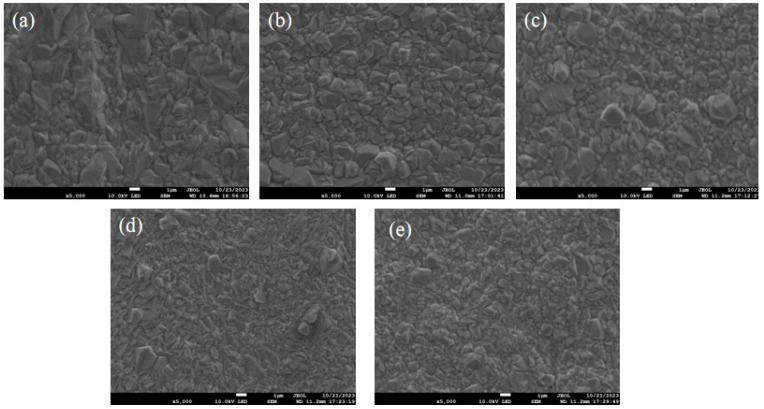
Surface morphology of the copper sheet plated with different concentrations of plating solution at SEM5000×. (**a**–**e**) The concentrations of SN2 are 0, 1, 2, 3, and 4 ppm, respectively.

To study the impact of additives on the quality of copper coatings, a scanning electron microscope (SEM) was employed to observe the surface morphologies of electroplated copper coatings with different additive contents at a magnification of 5000 times. From Figure 8a, the copper layer electroplated from the electroplating solution without the addition of SN2 has relatively large grains and shows a massive shape. As can be seen from Figure 8b–d, the grains on the plated surface after the addition of the organic additive SN2 became finer and smaller, and the grains reached their finest at 3 ppm SN2 (as shown in Figure 8d). The results indicate that organic additives are indispensable components in the electroplating solution. The addition of 2-mercapto-1-methylimidazole can improve the surface morphology, and the optimal effect is achieved at a concentration of 3 ppm.

**Figure 9:**
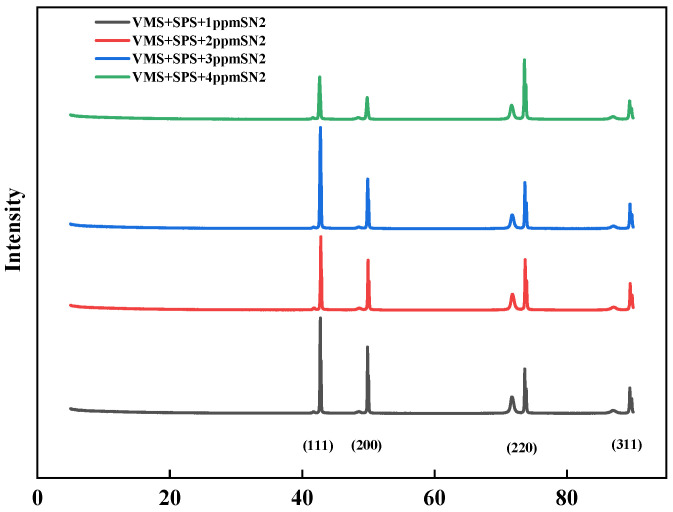
X-ray diffractograms of copper layers plated in plating solutions with different concentrations of levelers.

Figure 9 shows the XRD spectra of electroplated copper sheets in electroplating solutions with different concentrations of levelers. The orientated peaks are at about 43°, 51°, and 74°, corresponding to Cu (111), Cu (200), and Cu (220), respectively [22]. The stronger the orientation of the copper 111 crystal plane, the denser its structure [23]. The base bath is dominated by copper (220) facets, and (111) and (200) facets dominate after adding the additive SN2. As the leveler concentration increases, the selective growth of the electroplated copper layer along the (220) and (200) crystal planes weakens, while that along the (111) crystal plane strengthens, with the (111) crystal plane being the strongest in copper at 3 ppm. This implies that the leveler concentration significantly affects the optimal growth texture of the electroplated copper layer. In conclusion, the studied levelers can enhance the growth of the copper surface in the optimum orientation at appropriate concentrations.

There may be selective growth differences in copper due to the difference in depositional environments at the pore mouth and bottom. To further compare the preferential growth of copper at the pore opening and the pore bottom, X-ray diffraction was used to analyze the structure of the copper layer in the high-convection, high-current region (corresponding to the copper plating at the pore mouth) and the low-convection, low-current region (corresponding to the copper plating at the pore bottom).

**Figure 10:**
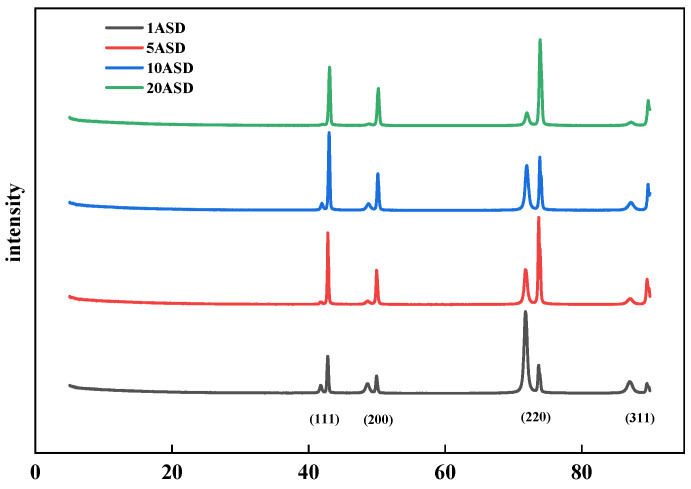
X-ray diffractograms of copper layers plated at different current densities.

Figure 10 shows the XRD patterns of copper layers electroplated at different current densities. As the current density increases, the intensity of the 111 diffraction peaks first increases and then decreases. The effect is better at 10 ASD, when the intensity of the 220 diffraction peaks is the smallest. However, after the current density is increased to 20 ASD, the 220 diffraction peaks dominate, and an overly high current density will disrupt the effect of SN2 on the crystal shape. During the electroplating process, there are differences in concentration and current intensity in the environments at the pore opening and the pore bottom. This leads to different adsorption conditions of the leveler at these two locations, which in turn results in differences in the crystal orientations of the electroplated copper layers at the pore opening and the pore bottom.

### 3.4. Theoretical Calculation Analysis

#### 3.4.1. Quantum Chemical Calculations

**Figure 11:**
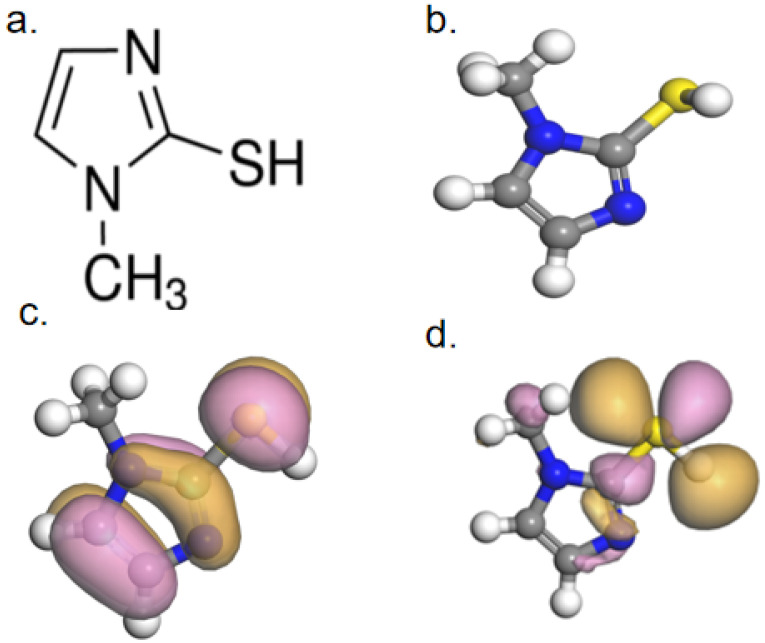
(**a**) Molecular structure; (**b**) optimized molecular structure; (**c**) HOMO distribution; and (**d**) LUMO distribution.

Quantum chemical calculations can clearly reveal the inherent correlation between the leveling effect of levelers and their molecular structures. The optimized molecular structure of SN2 and the electron density cloud distribution of the frontier molecular orbitals were obtained through quantum chemical calculations. The relevant parameters are shown in Figure 11. In it, yellow represents sulfur atoms (S), blue represents nitrogen atoms (N), grey represents carbon atoms (C), and white represents hydrogen atoms (H).

Figure 11a,b displays the initial and optimized molecular structures of 2-mercapto-1-methylimidazole, respectively. The distributions of the molecule’s LUMO and HOMO are shown in Figure 11c,d, respectively. The HOMO distribution is relatively uniform, spreading over both the heterocyclic ring and the mercapto group. This also indicates that SN2 molecules can be closely adsorbed onto the copper surface by donating the lone-pair electrons of these groups to the empty orbitals of copper. In contrast, the LUMO of SN2 is mainly located on the sulfhydryl group. The calculated E_LUMO_ value for 2-mercapto-1-methylimidazole is −0.667 eV, the E_HOMO_ value is −4.485 eV, and the energy gap ΔE is 3.82 eV. Generally, E_HOMO_ characterizes the strength of an organic molecule’s ability to provide electrons, while E_LUMO_ characterizes its ability to accept electrons. Usually, if an organic substance has a high E_HOMO_ value and a low E_LUMO_ value, it indicates a strong adsorption capacity on the metal surface. In other words, the smaller the ΔE value, the stronger the adsorption capacity of the organic molecule on the metal surface. The fact that the ΔE value of SN2 is only 3.82 eV demonstrates its strong adsorption capacity on the Cu surface, implying that the organic molecule can block Cu deposition on the pore surface during the electroplating process.

#### 3.4.2. Molecular Dynamics Simulation

**Figure 12:**
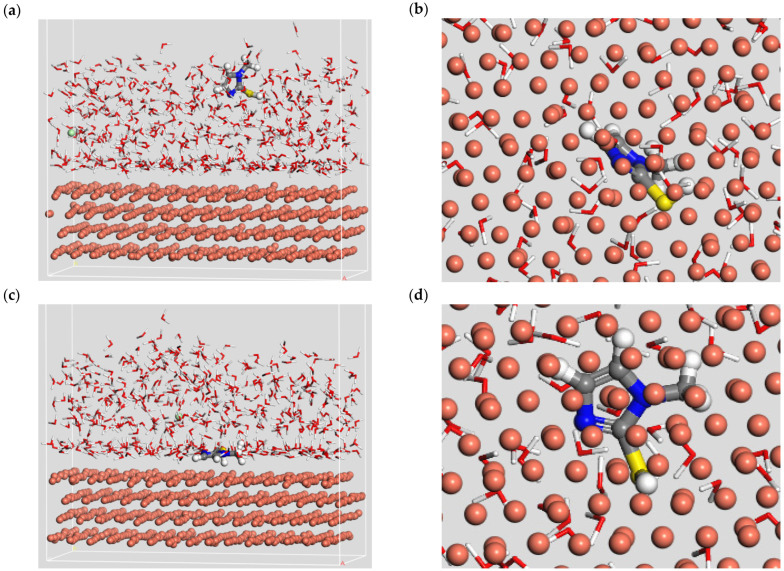
(**a**,**b**) Front and top views of the initial state of SN2 molecules adsorbed on the copper surface; (**c**,**d**) front and top views of the equilibrium state of SN2 molecules adsorbed on the copper surface.

Molecular dynamics simulation can analyze the motion state of leveler molecules through simulation trajectories, thus clarifying the interaction mode between the leveler and the metal copper surface. With the help of the Materials Studio software, the motion of the leveler SN2 on the copper (111) crystal plane was analyzed. As shown in Figure 12, at the adsorption equilibrium state of 500 ps, 2-mercapto-1-methylimidazole adsorbed on the copper surface in a unique parallel posture, which increased the contact area between the leveler and copper, weakened the deposition of copper on the surface, and the adsorption energy was −238.67 kJ/mol. Through the above calculations and simulations, the adsorption behavior of 2-mercapto-1-methylimidazole is theoretically demonstrated to have an inhibitory effect.

#### 3.4.3. XPS Experiment

**Figure 13:**
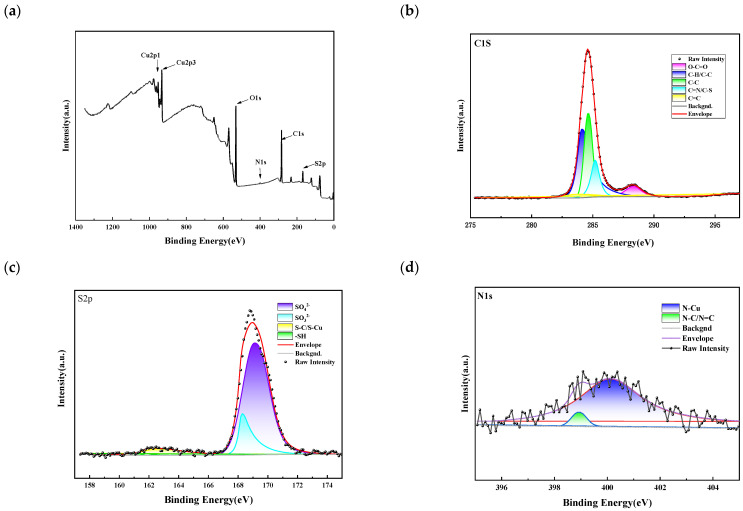
(**a**) Full spectrum; (**b**) C1s; (**c**) S2P; and (**d**) N1s. The red color line represents the peak corresponding to N-Cu after fitting, the filled peak below is purple.

Figure 13 shows the experimental results of 2-mercapto-1-methylimidazole XPS. C1s spectral decomposition resulted in five peaks corresponding to different carbon environments. The peaks at 238.6 eV, 284.1 eV, 284.5 eV, 285.2 eV, and 287.9 eV correspond to C = C, C-H/C-C, C-C, C = N/C-S, and O-C = O, respectively, indicating that the C element in the SN2 molecule is present on the copper surface. The S2p spectrum decomposes into four peaks located at bond energies 161.8 eV, 162.6 eV, 168.2 eV, and 169.1 eV, correspond to -SH, S-C/S-Cu, SO_3_^2−^, and SO_4_^2−^, respectively, where ¬SH may originate from the sulfhydryl group in SN2, and its presence with the rest of the S species further suggests that SN2 reacts with the copper surface in a relevant way. The N1s peak is decomposed into two peaks: one at bond energy 399 eV, which corresponds to N-C/N = C, and the other at 400.5 eV, which corresponds to N-Cu. The results of XPS combined with MD simulation indicate that the thiol group (-SH) in the SN2 molecule and the nitrogen atoms of the imidazole ring are the main adsorption sites. The plane of the imidazole ring is close to the copper surface, allowing the lone-pair electrons of the nitrogen atoms to coordinate with copper, thus hindering the deposition of copper ions and making the copper surface smooth. Other parts, such as the methyl group and the carbon atoms in the imidazole ring, interact with the surface only through van der Waals forces. Therefore, the C/S-Cu bond signals in XPS are weak or absent.

## 4. Conclusions

In this experiment, 2-mercapto-1-methylimidazole, a small molecule compound containing a sulfur-azepine ring, is proposed as a novel leveler. It enables bottom-up super-filling of blind vias without inhibitors. The results of the cathodic polarization and galvanostatic measurements indicate that an increase in the concentration of 2-mercapto-1-methylimidazole significantly enhances cathodic polarization and efficiently hinders copper deposition at the orifice and on the surface during electrodeposition. Moreover, electrochemical tests indicate that the concentration of chloride ions has a significant impact on the ability of SN2 to inhibit copper electrodeposition. With the addition of the leveler SN2, the sample surface changes from rough to smooth, the grains become finer, and the flatness improves greatly. The combination of theoretical calculations and XPS results shows that the thiol group (-SH) in the SN2 molecule and the nitrogen atoms of the imidazole ring are the main adsorption sites. The plane of the imidazole ring is adsorbed on the copper surface in a unique parallel posture, enabling the lone-pair electrons of the nitrogen atoms to coordinate with copper. This increases the contact area between the leveler and copper, thereby hindering the deposition of copper ions and making the copper surface smooth. In summary, the 2-mercapto-1-methylimidazole molecule is a potentially effective leveler for bottom-up super-filling of wafers.

## Data Availability

The original contributions presented in this study are included in the article/Supplementary Materials. Further inquiries can be directed to the corresponding author.

## Supplementary Materials

The following supporting information can be downloaded at https://www.mdpi.com/article/10.3390/ma18071622/s1, Experimental Method S1: Electrochemical measurement process.
