# Supplementary material for: Investigation of 2-Mercapto-1-Methylimidazole as a New Type of Leveler in Wafer Electroplating Copper
Source: Materials (Basel). 2025 Apr 2;18(7):1622. doi: 10.3390/ma18071622 (PMC11990726; doi:10.3390/ma18071622)
Supplement: Supplementary file 1 [file materials-18-01622-s001.zip › materials-3527120-supplementary.pdf]

Support material S1

All electrochemical experiments were carried out at room temperature. Before each measurement, the rotating disk electrode Pt-RDE was placed in the basic plating solution, and a thin copper layer with a thickness of approximately 500 nm was plated at a fixed rotation speed of 1000 rpm to convert Pt-RDE into Cu-RDE. In this study, the voltage scan range of the cyclic voltammetry (CV) experiment was set between 0.4 V and -0.8 V, and the scan rate was kept constant at 50 mV/s. The voltage scan range of the cathodic polarization curve was from 0 V to -1 V, and the scan rate was constantly maintained at 10 mV/s. The galvanostatic measurements (GMs) were conducted under a current density of 2 A/dm^2, and various additives were continuously added during the measurement process. The rotation speeds of the working electrode were fixed at 100 rpm and 1000 rpm, respectively, to simulate the low-current region at the bottom of the blind hole and the high-current region on the surface of the blind hole.

The Virgin Makeup Solution (VMS) used consists of 220 g/l of copper sulphate pentahydrate mixed with 55 g/l of sulfuric acid. The Cl- used in the experiment was 50 mg/l and the SPS was 1 mg/l.

Electrodeposition

The cut wafer (2 × 2 cm) was immersed in ethanol for 5 min to remove the

contaminants from the slices, and then rinsed with deionized water to ensure that the contaminants were rinsed clean to obtain ethanol pre-treated slices. The pre-treated wafer slices were immersed in electroplating solutions containing different concentrations of additives for 10 min. With the 2 × 2 cm wafer slice being used as the cathode and the phosphorus-containing copper plate being used as the anode, and with the magnetic stirring speed being set at 400 rpm, direct current was applied to the cathode and anode, and 1 ASD was plated for 60 s, followed by 5 ASD for 300 s, and then 10 ASD for 1 hour.
